# Portable nanoporous electrical biosensor for ultrasensitive detection of Troponin-T

**DOI:** 10.4155/fso.15.22

**Published:** 2015-11-01

**Authors:** Nandhinee Radha Shanmugam, Anjan Panneer Selvam, Thomas W Barrett, Steven C Kazmierczak, Milin Nilesh Rana, Shalini Prasad

**Affiliations:** 1 Department of Bioengineering, University of Texas at Dallas, Richardson, TX, USA; 2 Department of Veterans Affairs, Oregon Health & Science University, Portland, OR, USA; 3 Department of Pathology, Oregon Health & Science University, Portland, OR, USA; 4 Department of Molecular & Cell Biology, University of Texas at Dallas, Richardson, TX, USA

**Keywords:** electrical double layer, impedance spectroscopy, nanoconfinement, nanoporous, Troponin-T

## Abstract

**Aim:**

To demonstrate the design, fabrication and testing of a portable, label-free biosensor for ultrasensitive detection of the cardiac Troponin-T (cTnT) from patient blood.

**Materials & methods:**

The biosensor is comprised of a nanoporous membrane integrated on to a microelectrode sensor platform for nanoconfinement effects. Charge perturbations due to antigen binding are recorded as impedance changes using electrochemical impedance spectroscopy.

**Results:**

The measured impedance change is used to quantitatively determine the cTnT concentration from the tested sample. We were successful in detecting and quantifying cardiac Troponin-T from a 40-patient cohort. The limit of detection was 0.01 pg/ml.

**Conclusion:**

This novel technology has promising preliminary results for rapid and sensitive detection of cTnT.

## Abstract (lay abstract)

Quantification of biomarkers is essential for disease diagnosis and management. Current laboratory-based analytical methods are labor-intensive and rely mostly on use of labels for detection. A simple, point-of-care method based on a label-free technique offers robust real-time measurements for detection of biomarkers. This study intended to develop a nanoporous electrical biosensor to measure the cardiac biomarker cardiac Troponin-T. A nanotechnology approach towards sensor design improves detection sensitivity. The sensor performance for detection of cardiac Troponin-T demonstrates the potential for adoption in a clinical setting. However, further validation experiments are required prior to implementation.

**Figure 1.**
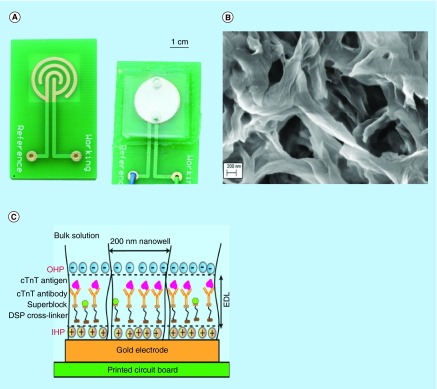
**Components of the developed sensor platform.** **(A)** Assembled sensor platform. **(B)** SEM micrograph of nanoporous nylon membrane with average pore diameter of 200 nm. **(C)** Characteristic representation of immunoassay inside nanowells. cTnT:Cardiac Troponin-T; DSP:Dithiobis succinimidyl propionate; EDL:Electrical double layer; IHP:Inner Helmholtz plane; OHP:Outer Helmholtz plane.

**Figure 2.**
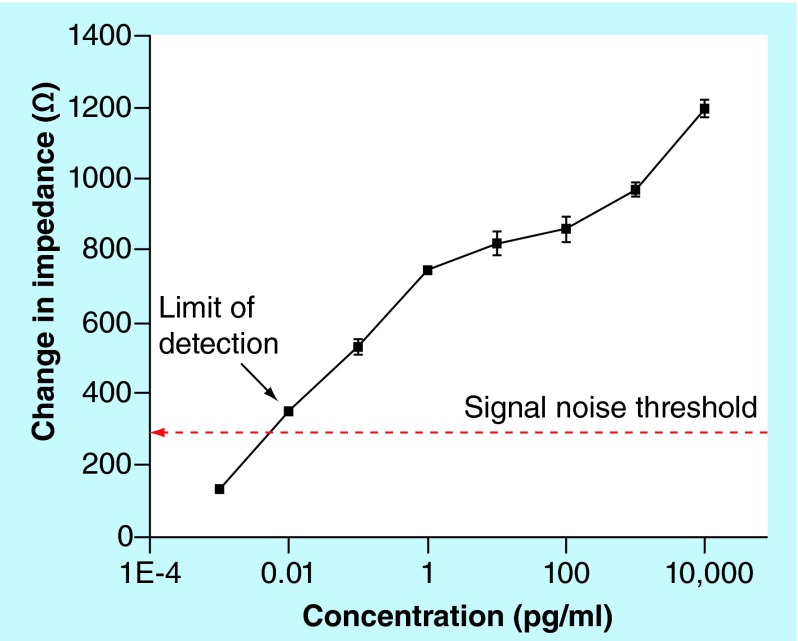
**Calibration dose response analysis for cardiac Troponin-T antigen diluted in 7% bovine serum albumin.** Cardiac Troponin-T concentrations in the range of 0.001 pg/ml to 10,000 pg/ml were analyzed for n = 3 replicates. Error bars indicate two standard deviation for n = 3 replicates.

**Figure 3.**
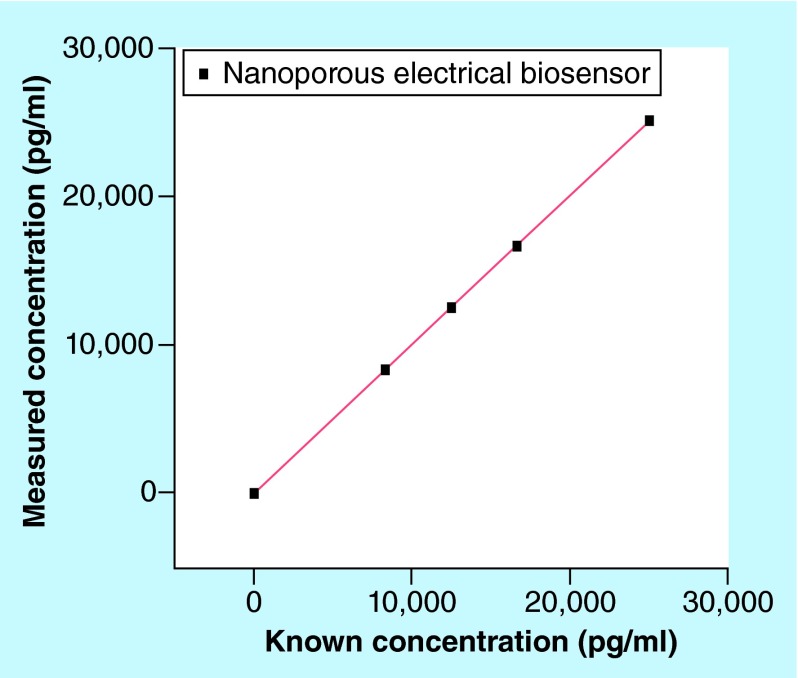
**Regression plot for linearity experiments.**

**Figure 4.**
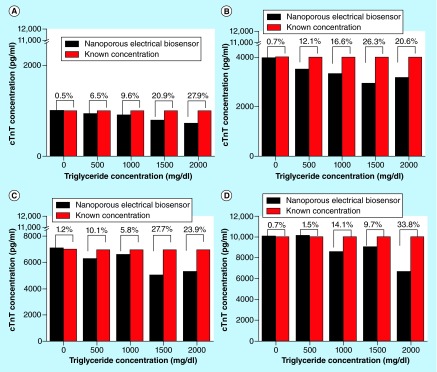
**Interference experiments with triglyceride concentrations in the range of 0 mg/dl–2000 mg/dl.** Triglyceride aliquots with cTnT concentration of **(A)** 1000 pg/ml; **(B)** 4000 pg/ml; **(C)** 7000 pg/ml and **(D)** 10,000 pg/ml. cTnT:Cardiac Troponin-T.

## Introduction

Measurements of cardiac Troponin-T (cTnT) and Troponin-I (cTnI) are superior to conventional measurement of creatine kinase isoenzyme MB (CK-MB) for the detection of myocardial infarction [1–4] and are valid predictors of adverse events in patients with acute coronary syndromes [2–3,5]. However, the point-of-care (POC) devices for measuring cardiac biomarkers have not kept up with laboratory-based methods. The laboratory-based analytical devices are much more sensitive than the POC devices, so are preferentially used for diagnosing heart attack [6]. In this study, preliminary performance characteristics of a novel nanoporous electrical biosensor for detection of cTnT were compared with a standard chemiluminescent laboratory-based assay. Electrical detection techniques that convert protein interactions into measurable signal could improve the speed, accuracy and reliability for measuring protein biomarkers [7–9]. Hence, in this paper we present a novel sensing platform using label-free electrochemical method for sensitive detection of cTnT from patient blood. The sensor platform discussed here consists of a microelectrode integrated with nanoporous membrane to provide size-based confinement for biomolecules [10,11]. Protein biomarker binding events occurring in the confined spaces consequently perturb the electrical properties of the microelectrode. The resulting electrical response is used to quantify proteins for clinical diagnosis.

The underlying phenomenon of our work is the use of a nanoporous membrane that forms the basis for macromolecular crowding and confinement. Inside cells, the ensemble of macromolecules participate in intracellular biochemical activity and there exists steric repulsions amongst them [12,13]. The presence of repulsive interactions restricts the free movement of proteins in the cellular environment and functionality in a highly crowded fluid media. Protein movement restricted to a fixed or impenetrable boundary is termed macromolecular confinement or nanoconfinement [14,15]. In our approach, a nanoporous membrane with high density of well-defined nanowell arrays offers an excluded-volume effect, in other words, volume exclusion due to presence of a protein molecule and no second molecule can occupy the same space [16]. These effects reduce signal noise due to nonspecific interactions and regulate the reaction kinetics and equilibrium of protein binding in physiological buffer medium. Thus, entropic binding of proteins due to crowding and nanoconfinement are leveraged to systematically quantify the increased biomarker activity in the nanowells under steady state conditions.

The target biomarker interacts with a capture probe that is immobilized on the sensing region and is then converted into a detectable signal. A detection technique that uses labels/tags interferes with inherent binding properties of biomolecules [9,17–18]. Labeling increases the noise signal from nonspecific interactions and has difficulties integrating with miniaturized POC devices [19]. Label-free methods are promising because they enable faster detection with greater accuracy with miniaturized hardware [20,21]. In our design, the target biomarker interaction at the sensing electrode occurs within the electrical double layer (EDL). These interactions perturb the charge distribution at electrode surface and are characterized using electrochemical impedance spectroscopy (EIS) [22,23]. In EIS, a small sinusoidal AC voltage signal under constant bias is applied to the electrodes over a single/wide frequency range and the resulting current is measured. EIS can be used to monitor the impedance (ratio between current and voltage) or capacitance changes at the electrode interface. In short, EIS-based POC biosensors have potential for simple and rapid quantitative measurement of biomarkers for diagnosis and monitoring of disease.

The developed sensor platform was tested for detection of the cardiovascular biomarker, cTnT, and compared with the Elecsys 4th generation cTnT assay (Roche Diagnostics, IN, USA), which is based on chemiluminescence and has a limit of detection at 10 pg/ml and detection time of 18 min [24,25].

## Materials & methods

### Device design

The biosensor platform consists of a gold microelectrode electroplated on to a FR-4 printed circuit board. A concentric interdigitated electrode pattern with an interspacing distance of 1 mm was chosen to minimize field interferences with signal measurements due to the presence of edges. The counter and reference electrodes were combined as one electrode and the total surface area of the microelectrode was estimated to be 135 mm^2^. Two gold electrodes, one as a working and other as a counter/reference electrode with 10-µm thickness each were used for electrochemical measurements. Surface area of working to reference electrode was in the ratio of 1:10. A nanoporous nylon membrane with 45–50% porosity was laid over the microelectrode to provide size matching for protein biomarker interaction and nanoconfinement. The arrays of confined nanoscale wells in the nylon membrane have pore a diameter of 200 nm. These nanoscale confined spaces act as a filter for larger molecules which would otherwise cause nonspecific interactions. The total surface area of the nanowell biosensor was calculated to be approximately 1.89 × 10^10^ mm^2^ considering that the membrane has approximately 500 million nanowells. A microfluidic encapsulant made of biocompatible polydimethylsiloxane (PDMS) was bonded to the FR-4 board to hold samples at the sensing regions. The encapsulant has inlet and outlet microfluidic ports that can accommodate a 100 µl sample volume. In its entirety, the developed sensor platform (shown in Figure 1A) offers nanoconfinement (Figure 1B & Figure 1C) effects for sensitive detection of cTnT.

### Principle of transduction method: EIS

Electrochemical impedance spectroscopy (EIS) is a technique used to characterize the impedance of an electrochemical system. In EIS, the impedance is determined by applying a small excitation sinusoidal AC voltage signal over range of frequencies at constant DC bias. The output is a phase shifted current signal in the same frequency domain. The ratio of voltage to the resulting current determines the electrochemical impedance. The measured impedance has real (Z_real_) and imaginary (Z_imag_) components as described in the following complex equation:Equation 1
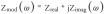



In the above equation, the total impedance (Z_mod_) is expressed as a function of radial frequency (ω). By determining the time constants of measured signal, it can be used to determine electronic/ionic conduction at the electrode surfaces, diffusion profile of molecules, charge-transfer and changes at the EDL [26,27]. In our work, we have used EIS to characterize dynamic changes occurring within EDL due to binding of cTnT. When a liquid is added to the sensor, the ions get adsorbed to charged electrode surface by electrostatic forces. A transfer of charges between the electrode and ions in the electrolyte occurs in order to maintain equilibrium, giving rise to charge-transfer resistance (R_ct_). At each step of the immunoassay, charge separation occurs within EDL due to binding events which occur in each nanowell. These charge perturbations within nanowell results in changes to double layer capacitance (C_dl_) that are reflected Z_imag_. The bulk medium contains higher concentrations of protein biomarkers and ions which offer some resistance to diffusion of molecule to electrode surface and are termed solution resistance (R_s_). These resistive components can be obtained from Z_real_. The described electrical parameters can be obtained by fitting the measured impedance data to Randel's equivalent circuit [27]. In our work, impedance measurements were taken by applying 10 mV sinusoidal signal at zero DC bias over 50 to 1200 Hz frequency range. Use of redox probe is eliminated in our sensor design so as to achieve robust measurements through nonfaradic biosensing.

### Immunoassay protocol for nanoporous electrical biosensor

The first step in the immunoassay was to functionalize the gold microelectrodes with a cross-linker molecule. Due to stability of gold–sulfur covalent bond [28], the sensing site was immobilized with a 10 mM solution of dithiobis succinimidyl propionate (DSP; Pierce Biotechnologies, IL, USA), a thiol cross-linker dissolved in dimethyl sulfoxide (DMSO). Following a 30 min incubation, excess DSP is removed using a DMSO wash. The sensing site was then incubated with antibody for another 30 min followed by buffer wash. The antibody concentration was chosen so as to have maximum surface coverage and was identified through antibody saturation experiments done previously [29]. A 100 ng/ml solution of affinity purified monoclonal antitroponin-T antibody (US Biological, MA, USA) dissolved in phosphate buffered saline (PBS; Thermo Scientific) was added to the sensor. The amine reactive NHS (N-hydroxysuccinimide) ester group at the end of DSP's 120-nm spacer arm interacts with the primary amine group of the antibody through an amine bond. To block the unoccupied DSP sites and thus eliminate nonspecific protein interactions, the sensor was then treated with Superblock (Thermo Scientific, MA, USA) for 15 min. The inoculated sensor platform was washed thrice with PBS to remove any unbound molecules. The impedance measurement at this step corresponds to baseline/zero dose response of the sensor. Finally, the test sample containing aliquot/unknown concentration of protein biomarker, cTnT was added to sensor and incubated for 15 min. The impedance change from the baseline measurements was determined for quantification of cTnT. Impedance measurements at each above mentioned immunoassay step were taken using Gamry Reference 600 potentiostat (Gamry Instruments, PA, USA). The sample volume of 100 µl was used for these measurements.

### Design of experiments

#### Calibration dose response

To build the calibration curve for detection of cTnT, the Superblock treated sensor was first treated with 7% bovine serum albumin (BSA; Sigma-Aldrich, MO, USA) solution containing zero concentration of protein biomarker. A 7% BSA was chosen as the diluting buffer medium to avoid any unmeasured Troponin-T that may have been present in commercial Human Serum and to simulate the physiological environment. Serial dilutions of purified cTnT antigen (US Biological) in concentrations of 10,000 pg/ml, 1000 pg/ml, 100 pg/ml, 10 pg/ml, 1 pg/ml, 0.1 pg/ml, 0.01 pg/ml and 0.001 pg/ml were prepared by diluting stock antigen in 7% BSA. Antigen concentrations, starting with the lowest concentration, was added to the sensing surface and allowed to incubate at room temperature for 15 min. The troponin T antibody (US Biological) recognizes epitopes between amino acids 60 and 70. Impedance measurement was taken thrice at each antigen doses. The nanoporous electrical biosensor was washed three-times with 7% BSA between subsequent doses. The change in impedance from baseline measurements was determined to build the calibration dose response curve.

#### Clinical sample collection

Remainder, discard heparinized plasma was collected from 40 patients with suspected myocardial infarction between September 2013 and February 2014. Samples were stored and maintained at negative 80°C until analysis. The patient samples were analyzed in duplicate on our nanoporous electrical biosensor platform to assess the accuracy and repeatability of our device. The results were compared with the Elecsys 4th generation Troponin-T assay (Roche Diagnostics). The demographic data and other clinical information on all the patients included in this study are unavailable. Though lack of patient information is a major shortcoming, the focus of this paper is to demonstrate ultrasensitive detection of Troponin-T using nanoporous electrical biosensors.

#### Linearity experiments

EIS measurements are typically measured by applying a small amplitude sinusoidal voltage signal, so the response of the system under study is pseudolinear. To measure the how linear and responsive the system is, purified cTnT antigen samples were diluted in a range of 0 pg/ml and 25,000 pg/ml (i.e.,0 pg/ml, 8330 pg/ml, 12,500 pg/ml, 16,670 pg/ml and 25,000 pg/ml), and were prepared in 7% BSA for n = 4 replicates.

#### Precision study

This study evaluated the reproducibility, accuracy and precision between measurements repeated at both the same and different time periods with the identical sample. Two purified quality control samples containing low (73 pg/ml) and high (1860 pg/ml) concentrations of cTnT were analyzed in duplicate for four consecutive days in both the morning and afternoon.

#### Functional sensitivity

Experiments were performed to measure the precision of measurements on the developed sensor platform at low concentrations of cTnT antigen. A known concentration of cTnT antigen of 600 pg/ml was used as original concentration. Eleven subsequent samples were prepared by diluting the sample with 7% BSA. The prepared concentration were 600 pg/ml, 300 pg/ml, 150 pg/ml, 75 pg/ml, 37.5 pg/ml, 19 pg/ml, 10 pg/ml, 5 pg/ml, 2.5 pg/ml, 1.25 pg/ml and 0.625 pg/ml. Each sample was analyzed on four different nanoporous electrical biosensor (n = 4 replicates). The purpose of the functional sensitivity experiment is to determine the limits of measuring cTnT for the technology.

#### Interference study

Testing for interferents is an important part of assessing the analytical specificity of new methods of measurement or technologies. We evaluated the potential interference from triglycerides (Intralipid; Sigma-Aldrich) with the nanoporous electrical biosensor's ability to detect cTnT. Four pools containing different concentrations of cTnT spiked with interferent substance were prepared. Purified Troponin-T antigen concentrations of 1000 pg/ml, 4000 pg/ml, 7000 pg/ml and 10,000 pg/ml in 7% BSA were spiked with triglycerides in varying concentrations between 0 and 2000 mg/dl. The prepared samples were then used on sensor platform and impedance measurements were taken for four replicates with each pooled concentrations (Table 1).

## Results & discussion

Figure 1B represents the SEM image of nylon membrane. As shown in the SEM micrograph, the pores are favorable for molecules to diffuse through to the electrode surface. The surface functionalization is another important parameter to determine the sensitivity and selectivity for detection of cTnT. As described earlier, DSP binding to the electrode surface within each nanowell forms a monolayer of tightly packed linker molecules. This layer not only provides binding sites for antitroponin-T specific to cTnT antigen but also chemical passivation to the electrode surface. The monolayer minimizes the interaction of ions/other molecules thus enhancing signal-to-noise ratio in nonfaradic EIS biosensors.

### Calibration study

The performance of nanoporous electrical biosensor was determined for varying concentrations of cTnT and the calibration curve was derived from 7% BSA as shown in Figure 2. The various components of the diluent/buffer medium impede flow of electric charges and hence contribute to resistance (Z_real_) in the observed impedance. The distribution of ions at the electrode surface results in formation inner Helmholtz plane (IHP) and outer Helmholtz plane (OHP). The biological molecules binding to the sensing sites occur within the fixed IHP layer and movable OHP layer, in other words, within EDL. The region of biomolecular binding resembles that of a parallel plate capacitor and hence results in capacitive changes observed as Z_imag_ in measured impedance. In the nanoporous electrical biosensor under study, the total impedance (Z_mod_) was used for determining the sensor performance. It was observed that as the binding of cTnT increases with increasing concentration of antigen doses, so that the impedance decreases due to dominant capacitive behavior. The change in impedance is determined by subtracting the impedance values between baseline (BSA wash step after Superblock step) and the antigen doses. The impedance change is directly correlated to the concentration of cTnT antigen. The dose response shown is average of three replicates. The measured value of impedance (Z_mod_), as shown in Figure 2, was used for estimating the sensor analytical performance. The signal noise of the system was determined as the impedance change between zero dose step and Superblock step. The limit of detection (LoD) was defined as the lowest concentration with impedance change greater than three-times the signal noise threshold. The observed impedance change was between 150 Ωs and 1195 Ωs with signal noise threshold determined at 298 Ωs. The sensor had overall linear dynamic range between 0.001 pg/ml and 10,000 pg/ml with LoD at 0.01 pg/ml in 7% BSA. The major focus of this study is to develop a diagnostic device capable of biomarker detection in a clinical setting. The developed platform have been tested and validated for known and established biomarker for cardiovascular disease detection, cTnT [2].

### Clinical sample analysis & validation

To clinically validate the sensor performance, the heparinized plasma from 40 patients with myocardial infarction was analyzed for presence of cTnT. The concentrations of cTnT in those samples were determined by fitting the observed impedance change to the calibration curve in Figure 2. A combined analysis of Z_mod_, Z_real_ and Z_imag_ as explained in previous section was followed so as to accurately estimate the concentration of cTnT. The concentration measured from the nanoporous electrical biosensor was compared with that obtained from the chemiluminescent Elecsys 4th generation assay. The immunoassay comparison for detection of cTnT is shown in Table 1. Regression analysis between these two different methods had an intercept of -4.132, 95% CI: -10.78–2.506; slope of 1.025, 95% CI: 1.016–1.034 and R-correlation value of 0.9998. Each sample was analyzed on two nanoporous electrical biosensor platforms. The coefficient of variation (CV) on our sensor platform was observed to be less than 4.5% which was well within the accepted clinical range of less than 20%.

The most sensitive cTnT method is defined as the method that detects the raised cardiac concentrations exceeding 99th percentile of the reference population (i.e., healthy population) with acceptable imprecision. Studies have shown that the 99th percentile is dependent on both the population screened and the device used to measure cTnT. A recent study used the same reference population of 525 presumably healthy patients (assessed by health questionnaire) and tested 19 troponin devices [6]. The highly-sensitive assays had a 2.5-fold variation in the 99th percentile, the contemporary devices had a 32-fold variation, and the POC devices had a fivefold variation. These data indicate, it is not possible to compare troponin values between devices. There is no guideline statement to define a normal, healthy reference population, although this has been suggested as an important next step. Reliable and sensitive devices that can measure cTnT concentrations at low levels are required to eliminate the controversies regarding 99th percentile Troponin-T estimation. However, future studies include investigating and establishing 99th percentile information in a healthy cohort using the developed nanoporous electrical biosensor.

### Linearity, precision & functional sensitivity

In order to validate the robustness of sensor, it is essential to perform precision studies that can demonstrate reproducibility and linearity in operation. The samples were analyzed twice in the morning and in the afternoon on individual nanoporous biosensor platforms for the precision study. The overall CV for a cTnT concentration of 72 pg/ml and 1860 pg/ml was 2.38 and 0.85%, respectively.

The sensor showed a linear response for all tested concentrations of cTnT between 0 pg/ml and 25,000 pg/ml with a CV of 2% (Figure 3). The regression analysis had an intercept at 15.88 pg/ml and Spearman's r correlation of 1.

The functional sensitivity of the immunoassay was identified at a CV of 10%. The concentration of cTnT was 0.33 pg/ml at a CV of 10%. Samples for functional sensitivity analysis were prepared with 7% BSA as diluent medium and each concentration was analyzed for n = 4 replicates.

### Interference analysis

The interference from the presence of triglycerides was observed to be not significant if triglyceride concentration was less than 500 mg/dl. Triglyceride concentration above 500 mg/dl had a CV greater than 10%. Figure 4 represents triglyceride as interferent for selected concentrations of cTnT. The data shown is average of n = 4 replicates.

These preliminary results indicate the electrical biosensor platform's capability to detect low concentrations of cTnT from patient blood samples, in a POC device with results available in 15 min. If a POC cTnT device were as sensitive and reliable as the laboratory-based analytical devices, then the turnaround time for cTnT results would improve. Most emergency departments do not meet the suggested turnaround time for cardiac troponin of 60 min [30,31]. A POC cTnT assay that would produce accurate results at the bedside would accelerate patient care as the results would be available for the clinician sooner, so treatment decisions could be made faster. In addition, a reliable POC cTnT device would enable improved triage in areas outside the emergency department such as doctors’ offices. The above described nanoporous electrical biosensor could satisfy these needs. In our sensor, each of the nanowells acts as individual sites for biomolecular binding and offers nanoconfinement. The phenomenon of macromolecular crowding maximizes the density of biomolecules in each of nanowells and hence increases the probability of cTnT antigen binding to antibody. Furthermore, combined measurement of binding events at each nanowell provides amplified signal output for detection of cTnT. The detection at low frequencies minimizes the effects of nonspecific interactions in the measured impedance signal.

The proposed method showed wide and linear dynamic range with good precision, reproducibility and linearity for detection of cTnT at ultralow concentrations. However, the performance of nanoporous electrical biosensors at high concentrations still remains to be addressed. The phenomenon of inter protein steric repulsion over a small sensing area and electrostatic noise from the solution resistance as well as buffer media remained dominant for high concentrations causing variability at high concentrations [32,33]. Improved sensor performance at high concentrations can be achieved by increasing the surface area of sensing sites as well as electrostatic nature by enhancing the porosity of nylon membrane or by use of different membranes with higher porosity. In this study, only few investigations have been performed using the developed platform for analysis of cTnT concentrations in complex patient samples. The variability at high concentrations can be improved by analysis of more complex samples with presence of cTnT and increasing signal-to-noise ratio of electrical biosensor. Hence, we demonstrate here only the proof-of-concept of nanoporous electrical biosensor as a potential alternative for ultrasensitive detection of cTnT. In short, this platform will open up new possibilities for clinical diagnosis of cTnT in a rapid, and simple manner.

## Conclusion

In this research, we have demonstrated the benefits of nanoconfinement within the nanowells for successful quantification of cardiac biomarker, Troponin-T at clinically relevant concentrations in patient blood. Incorporation of the nanoporous membrane enhances the sensitivity of detection with a limit of detection at 0.01 pg/ml in 7% BSA. These preliminary results demonstrate the potential of this platform to quantify cTnT in a clinical setting, with a turnaround time of 15 min.

## Future perspective

The demonstrated technology shows a road forward for portable and rapid point-of-care diagnostics using protein biomarkers. Future directions for advancing this technology will include testing with a larger cohort of patients experiencing chest pain, and completing Clinical and Laboratory Standards Institute (CLSI) standards. The rapid, reliable and portable measurement of very low levels of cTnT may enable all patients to know their baseline level of cTnT which may better risk stratify patients for future cardiovascular events at the personal level.

**Table 1.** **Table representing the comparison of cardiac Troponin-T concentrations between electrochemical and chemiluminescent immunoassay detection method.**

**Test sample**	**Electrochemical immunoassay (pg/ml)**	**Chemiluminescent immunoassay (pg/ml)**	**Variability between two methods (%)**
P1	0.61	<10	N/A
P2	70.85	68	2.9
P3	68	73	5.01
P4	79.8	81	1.06
P5	85.5	91	4.41
P6	92.65	94	1.02
P7	106.05	101	3.45
P8	90	102	8.84
P9	110.35	111	0.42
P10	112	112	0
P11	118.5	126	4.34
P12	116.5	127	6.1
P13	133.5	137	1.83
P14	154.65	153	0.76
P15	154.5	155	0.23
P16	156	167	4.82
P17	163	169	2.56
P18	195.9	199	1.11
P19	201.1	210	3.06
P20	255.9	259	0.85
P21	263	276	3.41
P22	284.4	284	0.1
P23	288.55	289	0.11
P24	312.65	315	0.53
P25	509.7	507	0.38
P26	508	517	1.24
P27	609.5	635	2.9
P28	671.5	686	1.51
P29	763.75	767	0.3
P30	771.5	782	0.96
P31	802.8	803	0.02
P32	837.25	821	1.39
P33	842.5	878	2.92
P34	981.5	911	5.27
P35	1075	1100	1.63
P36	1261.5	1120	8.4
P37	1222	1160	3.68
P38	1255	1310	3.03
P39	1765	1820	2.17
P40	2775	2840	1.64

Sensor performance was compared for n = 40 clinical samples.

**Executive summary**

A nanoporous electrical biosensor platform for cardiac Troponin-T (cTnT) detection in patient blood was designed and tested.
The developed platform has a detection limit at 0.01 pg/ml, much lower than commercially available immunoassays for cTnT.
The developed technology shows promising performance for detection of cardiac biomarker, cTnT, within 15 min.

## References

[1] Hetland O, Dickstein K (1998). Cardiac troponins I and T in patients with suspected acute coronary syndrome: a comparative study in a routine setting. *Clin. Chem.*.

[2] Hamm CW, Goldmann BU, Heeschen C, Kreymann G, Berger J, Meinertz T (1997). Emergency room triage of patients with acute chest pain by means of rapid testing for cardiac troponin T or troponin I. *N. Engl. J. Med.*.

[3] Qureshi A, Gurbuz Y, Niazi JH (2012). Biosensors for cardiac biomarkers detection: a review. *Sensors Actuat. B Chem.*.

[4] Newby LK, Jesse RL, Babb JD (2012). ACCF 2012 expert consensus document on practical clinical considerations in the interpretation of troponin elevations: a report of the American College of Cardiology Foundation task force on Clinical Expert Consensus Documents. *J. Am. Coll. Cardiol.*.

[5] Twerenbold R, Jaffe A, Reichlin T, Reiter M, Mueller C (2012). High-sensitive troponin T measurements: what do we gain and what are the challenges?. *Eur. Heart J.*.

[6] Apple FS, Ler R, Murakami MM (2012). Determination of 19 cardiac troponin I and T assay 99th percentile values from a common presumably healthy population. *Clin. Chem.*.

[7] Wan Y, Su Y, Zhu XH, Liu G, Fan CH (2013). Development of electrochemical immunosensors towards point of care diagnostics. *Biosens. Bioelectron.*.

[8] Nagaraj VJ, Aithal S, Eaton S, Bothara M, Wiktor P, Prasad S (2010). Nanomonitor: a miniature electronic biosensor for glycan biomarker detection. *Nanomedicine (Lond.)*.

[9] Luo XL, Davis JJ (2013). Electrical biosensors and the label free detection of protein disease biomarkers. *Chem. Soc. Rev.*.

[10] Xu K, Huang JR, Ye ZZ, Ying YB, Li YB (2009). Recent development of nano-materials used in DNA biosensors. *Sensors (Basel)*.

[11] Santos A, Kumeria T, Losic D (2013). Nanoporous anodic aluminum oxide for chemical sensing and biosensors. *Trends Anal. Chem.*.

[12] Minton AP (2001). The influence of macromolecular crowding and macromolecular confinement on biochemical reactions in physiological media. *J. Biol. Chem.*.

[13] Deng J, Toh C-S (2013). Impedimetric DNA biosensor based on a nanoporous alumina membrane for the detection of the specific oligonucleotide sequence of dengue virus. *Sensors (Basel)*.

[14] Zhou HX, Rivas GN, Minton AP (2008). Macromolecular crowding and confinement: biochemical, biophysical, and potential physiological consequences. *Annu. Rev. Biophys.*.

[15] Zaki A, Dave N, Liu JW (2012). Amplifying the macromolecular crowding effect using nanoparticles. *J. Am. Chem. Soc.*.

[16] Minton AP (2005). Models for excluded volume interaction between an unfolded protein and rigid macromolecular cosolutes: macromolecular crowding and protein stability revisited. *Biophys. J.*.

[17] Tsakalakis M, Bourbakis NG (26–30 August 2014). Health care sensor–based systems for point of care monitoring and diagnostic applications: a brief survey. *Engineering in Medicine and Biology Society (EMBC), 2014 36th Annual International Conference of the IEEE*.

[18] Pei-Wen Y, Che-Wei H, Yu-Jie H (2014). A device design of an integrated cmos poly-silicon biosensor-on-chip to enhance performance of biomolecular analytes in serum samples. *Biosens. Bioelectron.*.

[19] Grieshaber D, Mackenzie R, Voros J, Reimhult E (2008). Electrochemical biosensors – sensor principles and architectures. *Sensors (Basel)*.

[20] Daniels JS, Pourmand N (2007). Label-free impedance biosensors: opportunities and challenges. *Electroanalysis*.

[21] Di Capua R, Barra M, Santoro F (2012). Towards the realization of label-free biosensors through impedance spectroscopy integrated with ides technology. *Eur. Biophys. J.*.

[22] Macdonald MA, Andreas HA (2014). Method for equivalent circuit determination for electrochemical impedance spectroscopy data of protein adsorption on solid surfaces. *Electrochim. Acta*.

[23] Kumar RTK, Shanmugam NR, Prasad S (2013). Effect of size matching for ultrasensitive detection of protein biomarkers. *Nano LIFE*.

[24] Pagani F, Apple F, Garcia-Beltran L (2007). Results from a multicenter evaluation of the 4th generation Elecsys Troponin T assay. *Clin. Lab.*.

[25] Barrett TW, Radha Shanmugam N, Selvam AP, Kazmierczak SC, Prasad S (2015). Novel nanomonitor ultra-sensitive detection of troponin T. *Clin. Chim. Acta.*.

[26] Yang L, Bashir R (2008). Electrical/electrochemical impedance for rapid detection of foodborne pathogenic bacteria. *Biotechnol. Adv.*.

[27] Panneer Selvam A, Vattipalli KM, Prasad S (28 August–1 September 2012). Design of a high sensitive non-faradaic impedimetric sensor. *Engineering in Medicine and Biology Society (EMBC), 2012 Annual International Conference of the IEEE*.

[28] Laibinis PE, Whitesides GM, Allara DL, Tao YT, Parikh AN, Nuzzo RG (1991). Comparison of the structures and wetting properties of self-assembled monolayers of normal-alkanethiols on the coinage metal-surfaces, Cu, Ag, Au. *J. Am. Chem. Soc.*.

[29] Panneer Selvam A, Prasad S (2013). Nanosensor electrical immunoassay for quantitative detection of NT-pro brain natriuretic peptide. *Future Cardiol.*.

[30] Sandoval Y, Apple FS (2014). The global need to define normality: the 99th percentile value of cardiac troponin. *Clin. Chem.*.

[31] Apple FS, Simpson PA, Murakami MM (2010). Defining the serum 99th percentile in a normal reference population measured by a high-sensitivity cardiac troponin I assay. *Clin. Biochem.*.

[32] Minton AP (1993). Macromolecular crowding, confinement, stickiness, and the organization of cytoplasm. *Biophys. J.*.

[33] Minton AP (1994). Effects of macromolecular crowding on molecular recognition. *Biophys. J.*.

